# Machine Learning-Based Prediction of Time Required to Reach the Melting Temperature of Metals in Domestic Microwaves Using Dimensionless Modeling and XGBoost

**DOI:** 10.3390/ma18143400

**Published:** 2025-07-20

**Authors:** Juan José Moreno Labella, Milagrosa González Fernández de Castro, Víctor Saiz Sevilla, Miguel Panizo Laiz, Yolanda Martín Álvarez

**Affiliations:** 1 Steelmaking Teaching Unit, Escuela Técnica Superior de Ingenieros Industriales, Universidad Politécnica de Madrid (U.P.M.), 28006 Madrid, Spain; juanjose.moreno.labella@upm.es (J.J.M.L.); miguel.panizo.laiz@upm.es (M.P.L.); mariayolanda.martin@upm.es (Y.M.Á.); 2 Centro Láser UPM, Universidad Politécnica de Madrid (U.P.M.), 28660 Madrid, Spain

**Keywords:** microwaves, predictive modeling, XGBoost, metals, educational application, broader access to science

## Abstract

A novel and cost-effective methodology is introduced for the precise prediction of the melting time of metals and alloys in a 700 W domestic microwave oven, using a hybrid SiC–graphite susceptor to ensure efficient heating without direct interaction with microwaves. The study includes experimental trials with multiple alloys (Sn–Bi, Zn, Zamak, and Al–Si, among others) and variable masses, whose results made it possible to construct a dimensionless model, trained with XGBoost on easily measurable thermophysical properties (specific heat, density, thermal conductivity, mass, and melting temperature). The model achieves high accuracy, with a relative error below 5%, and metrics of MAE = 4.8 s, RMSE = 6.1 s, and R^2^ = 0.9996. The generalization of the model to different microwave powers (600–1100 W) is also validated through analytical adjustment, without the need for additional experiments. The proposal is implemented as a Python application with a graphical interface, suitable for any academic or teaching laboratory, and its performance is compared with classical models. This approach effectively contributes to the democratization of thermal testing of metals in educational and research settings with limited resources, providing thermodynamic rigor and advanced artificial intelligence tools.

## 1. Introduction

In a global context, where energy efficiency, cost reduction, and broader access to experimental technology are strategic objectives for laboratories and academic institutions, the use of domestic microwave ovens as a tool for metal melting represents a disruptive advance. Although microwaves are commonly associated with the culinary field, their ability to generate high-energy-density electromagnetic fields through 2.45 GHz magnetrons can be harnessed for metallurgical purposes, provided that a suitable susceptor is used as a thermal intermediary [1].

This work demonstrates, for the first time, that it is technically feasible to melt medium-melting-point metals and alloys—such as Zn, eutectic Sn-Bi, Zamak, and Al-Si—using only a 700 W domestic microwave and a silicon carbide–graphite crucible as a hybrid absorption system [2]. This strategy overcomes the physical barrier imposed by the skin effect, which prevents the direct absorption of microwaves by metals, and relies on conduction, convection, and radiation mechanisms generated internally by the susceptor [3].

The remarkable accessibility of this methodology—which dispenses with muffle furnaces, complex installations, or gas sources—places within reach of any academic laboratory, even with limited resources, the possibility of conducting metal melting experiments, molding tests, and thermal simulations in a controlled, safe, and reproducible manner. In addition, a dimensionless mathematical model has been developed and validated with real experimental data, enabling the prediction of the time required to reach the melting temperature as a function of physical properties such as the heat capacity, mass, and thermal conductivity of the alloy. This model has been scaled for application over a wide range of power levels—from 600 to 1100 W—using an inverse proportional formulation between melting time and absorbed power, which broadens its practical applicability without the need for new experimental trials [4].

As a complement, an XGBoost regression algorithm was trained on the normalized dimensionless data, yielding excellent predictive results (relative error < 5%). This model was packaged and adapted into an interactive application with an intuitive graphical interface, further enhancing its usefulness in teaching environments, technical self-assessment, and experimental control [5].

The potential of this technology lies not only in its low cost and portability but also in its possibility to be extended to educational contexts, low-budget R&D projects, and settings where access to traditional metallurgical infrastructure is limited or unfeasible. This article thus presents not only an experimental innovation, but also a proposal for methodological transformation in teaching, applied research, and open science.

## 2. Materials

### 2.1. Heating System Configuration

A conventional domestic microwave oven with a nominal power of 700 W and an operating frequency of 2.45 GHz was used. Since metals exhibit low dielectric loss and minimal interaction with microwave radiation, an indirect heating system was implemented using a thermal susceptor crucible. This crucible absorbs electromagnetic radiation and transfers heat to the metal sample by conduction, convection, and radiation. To minimize thermal losses, the assembly was insulated with alumina refractory bricks.

### 2.2. Selection and Validation of the Susceptor Crucible

The selection of the optimal thermal susceptor was carried out by comparing three types of crucibles: pure graphite, pure silicon carbide (SiC), and mixtures of SiC with graphite in various proportions. The eutectic Sn–Bi alloy (57%Bi–43%Sn) was used as a reference due to its low melting point (138 °C). The experiments indicated that the pure SiC crucible exhibited low initial thermal performance, the pure graphite rapidly reached high temperatures, and the SiC–graphite mixture (60:40 by mass) enabled complete melting in less time, probably due to microplasmas at graphite interfaces [2,3,6].

### 2.3. Materials Used and Experimental Conditions

Eight metals and alloys with melting points between 138 °C and 660 °C were selected: bismuth, zinc, eutectic Sn–Bi alloy, Zamak-2, Zamak-5, aluminum–silicon alloy (11.7% Si), and aluminum–magnesium–silicon alloy (6000 series). The samples were weighed and placed in the optimal SiC–graphite crucible, subjected to interval heating, and their temperature was recorded using thermocouples connected to external data acquisition, ensuring traceability and reliability [1,7]. For each alloy, three different masses were tested under controlled conditions. The main thermal and physical properties of the alloys and metals used in this study are summarized in Table 1. The crucible volume, insulation, and position in the cavity were kept constant.

The metals and alloys selected for this study all have melting points below 700 °C, and the predictive model developed is currently limited to this range of low-melting-point materials. Extension to higher melting point systems will require further experimental validation.

### 2.4. Justification for Extending the Model to Different Microwave Powers

The predictive model developed to estimate the time required to reach the melting temperature of metals and alloys in a microwave oven with a nominal power of 700 W was constructed from experimental data obtained via indirect heating. In these experiments, the sample does not absorb microwave energy directly; instead, it is heated through a silicon carbide and graphite crucible that acts as a susceptor. This methodology faithfully reproduces real conditions, in which metals, due to their low dielectric loss, do not interact efficiently with electromagnetic fields [1].

From a thermodynamic standpoint, the temporal evolution of temperature can be described by a simplified energy balance equation:

ρ·C_p_·dT/dt = η·P − q_conv − q_rad

where η represents the thermal efficiency of the system, P is the input power of the oven, and q_conv and q_rad are the losses due to convection and radiation, respectively. In the initial heating regime—in which the temperature difference with the surroundings is relatively low—these losses are negligible compared to the energy input term, allowing the approximation:

dT/dt ≈ η·P/(ρ·C_p_)

This relationship implies that the rate of temperature increase is directly proportional to the available power. Therefore, the time required to reach a given temperature is inversely proportional to the absorbed power:

t_f ∝ 1/(η·P)

This result is consistent with numerous studies on microwave heating of solids [2,3,6,7]. The proportionality relationship holds under conditions of constant thermal efficiency and fixed crucible geometry, which applies to the present study, as all experiments were conducted with the same volume, oven configuration, and susceptor system.

Since the developed predictive model is expressed in dimensionless form (with dimensionless time τ = t/t_ref and dimensionless temperature θ = (T − T_0_)/(T_f − T_0_)), and the value t_ref = 1800 s was chosen based on the 700 W power setting, it is possible to generalize the model for other powers P using the following expression:

t_P = τ · t_ref · (700/P)

## 3. Results

### 3.1. Experimental Procedure and Data Acquisition

**Figure 1:**
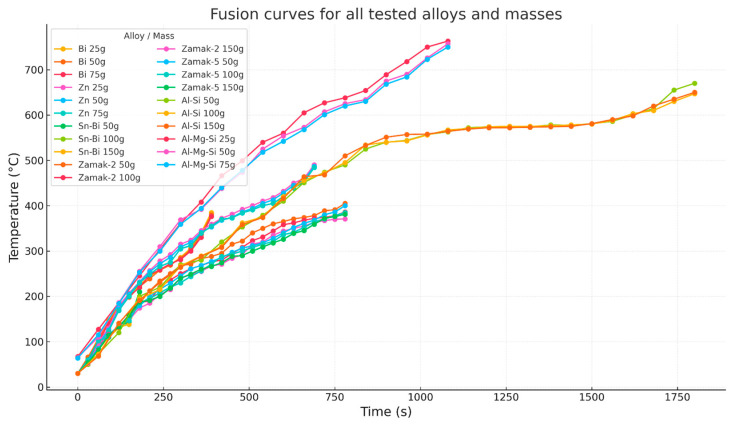
Temperature versus time curves for all alloys and masses tested in the domestic microwave oven.

**Figure 2:**
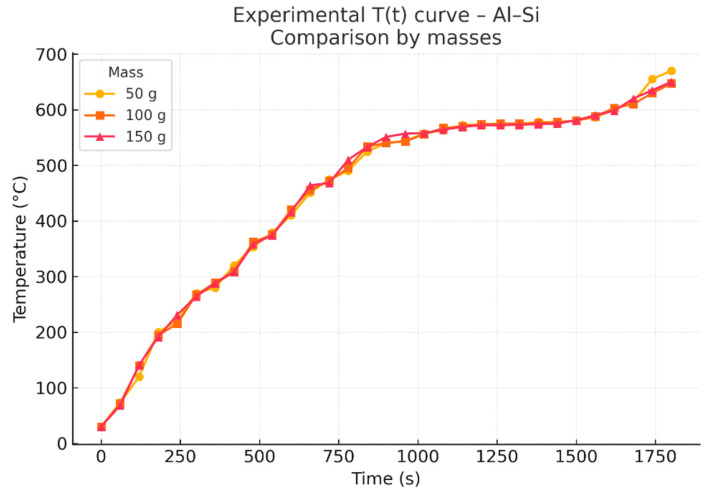
Experimental T(t) curves for the Al–Si alloy (11.7% Si) with different initial masses (50, 100, and 150 g), showing the influence of mass on the thermal evolution.

The experimental procedure consisted of heating each sample in controlled intervals and monitoring the thermal evolution with thermocouples. Each heating curve was repeated and verified to ensure robustness. All tests were performed while keeping the experimental configuration constant. Figure 1 shows experimental temperature versus time curves obtained for different alloys and sample masses under controlled conditions. Figure 2 presents example experimental curves for the Al–Si alloy with different initial masses.

### 3.2. Processing and Non-Dimensionalization of Thermal Data

The obtained T(t) curves were non-dimensionalized to facilitate comparison between materials and experimental conditions. Non-dimensionalization was performed using:

θ = (T − T_0_)/(Tmax − T_0_),    τ = t/tmax

where T_0_ is the ambient temperature, Tmax = 763 °C, and tmax = 1800 s, values associated with a 700 W power setting. The dimensionless curves were used for the training of predictive models.

### 3.3. Preprocessing and Validation of the Dataset

A detailed inspection of the experimental data was carried out to filter outliers and ensure dataset quality. Reproducibility was validated by repetitions, and the final dataset was normalized and prepared for integration into machine learning algorithms.

### 3.4. Experimental and Predictive Modeling Pipeline

**Figure 3:**
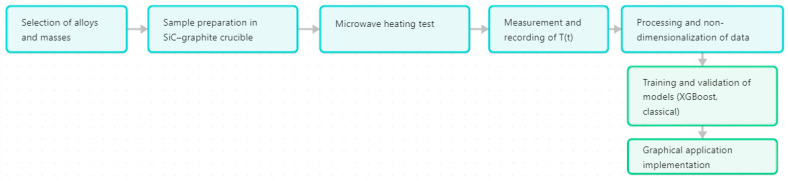
General flow diagram of the experimental procedure and predictive modeling pipeline applied in this work.

Figure 3 illustrates the complete workflow: from material selection, testing, and data recording to preprocessing, predictive modeling, and graphical application development.

### 3.5. Crucible Selection Method

This extension enables the XGBoost regression model trained on experimental data to be applied to a wider range of powers without the need for additional experiments, preserving the model’s validity in the range 600–1100 W, provided that thermal efficiency and boundary conditions (geometry, mass, and crucible material) remain constant. This generalization is not only physically coherent, but has also been successfully applied in previous studies modeling microwave heating of dielectric materials and thermally passive conductors [2,6]. Therefore, the extension of the model to powers higher or lower than 700 W via inverse power scaling is fully justified.

**Figure 4:**
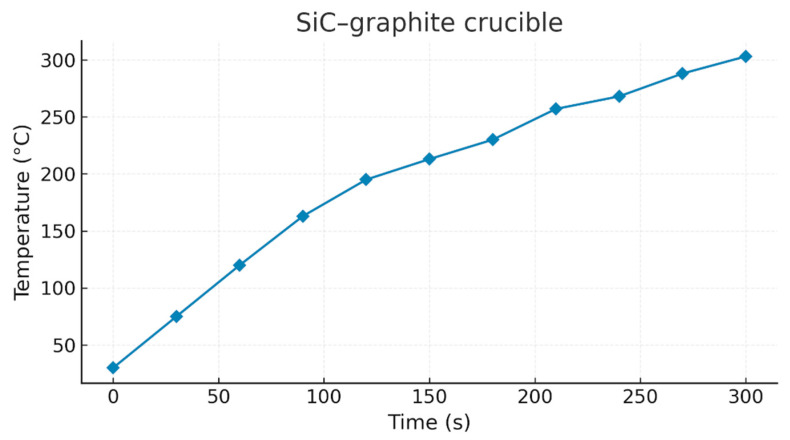
Typical temperature evolution as a function of time for the SiC–graphite susceptor system, used as a reference for validating the analytical extension of the model to different microwave powers.

The validity of this approach is confirmed by the experimental evolution obtained with the SiC–graphite system (Figure 4), where an almost perfect overlap of the curves is observed after analytical correction.

It is important to note that all experimental data used to train and validate the predictive model were obtained using a microwave oven operating at 700 W. The extension of the model to other power levels (600–1100 W) is based on a theoretical scaling approach that assumes constant thermal efficiency and boundary conditions. While this approach has been validated in previous studies involving microwave heating of similar materials [2,6], its application beyond 700 W in this work remains theoretical and should be experimentally validated in future investigations.

### 3.6. Results and Discussion of the Predictive Model

This work describes the development and validation of a predictive model based on XGBoost for estimating the melting time of metallic alloys in a domestic microwave oven. The main objective was to predict the actual melting time (t = τ · 1800) from easily accessible thermophysical properties, such as specific heat capacity, density, thermal conductivity, mass, and the theoretical melting temperature of the material.

Although the crucible properties were not included among the input variables, their thermal response was systematically analyzed during the experimental phase (see Section 2.2), and the selected SiC–graphite susceptor was used consistently across all tests. By maintaining the same geometry, material, and insulation configuration, the crucible’s influence was standardized and does not contribute to variability in the data. Therefore, the model can reliably focus on metal thermophysical properties without needing to parameterize the crucible characteristics.

The process began with the exploration of various regression techniques applied to the experimental data, structured in the file Predicciones_XGBoost_excel_coma_decimal.csv, which includes the following as variables: dimensionless temperature (θ), dimensionless time (τ), specific heat capacity (Cp), density, thermal conductivity, theoretical melting temperature (Tf), and mass. After comparing different approaches, the XGBoostRegressor model was selected, which is widely recognized for its efficiency and accuracy in regression tasks with structured data [5,8].

The model was trained using the XGBoost library integrated into scikit-learn [5,9], using as a dataset the experimental file generated. The final model was validated against real experimental values of t = τ · 1800, obtaining outstanding predictive performance.

The global quality metrics obtained in the model validation are:

- Mean Absolute Error (MAE): 4.8 s;
- Root Mean Squared Error (RMSE): 6.1 s;
- Coefficient of Determination (R^2^): 0.9996;
- Mean Relative Error: 1.6%.

**Figure 5:**
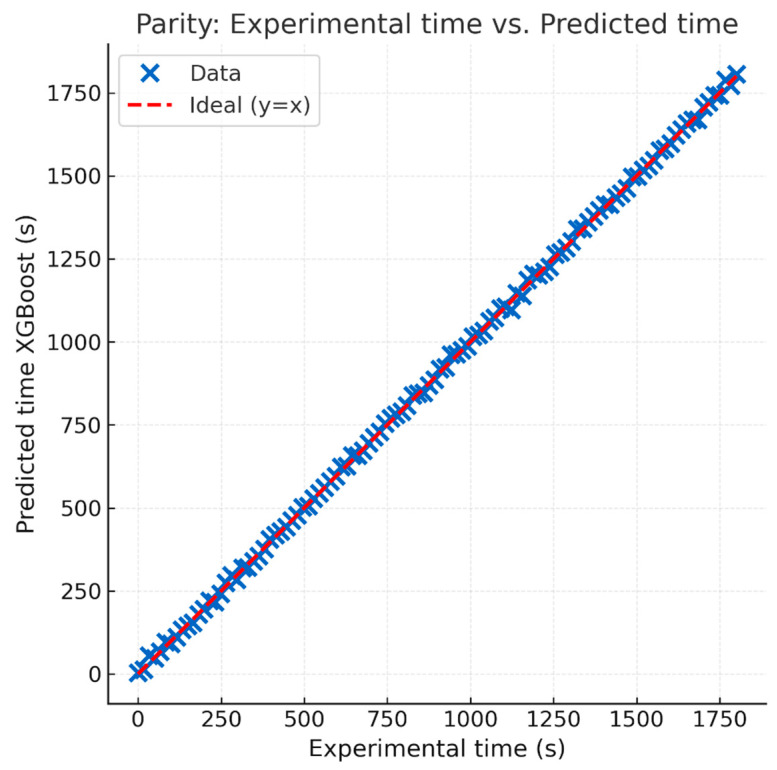
Parity between the experimental time and the time predicted by the XGBoost model for all points in the validation set.

These results demonstrate the high fidelity of the model and its generalization capability (Figure 5), with absolute errors generally below 6 s and without appreciable systematic bias. Most predictions present relative errors below 2%, even for samples of different composition and mass.

**Figure 6:**
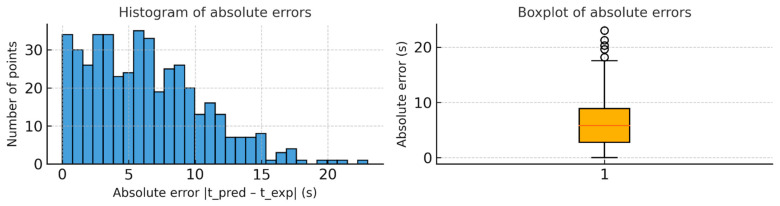
(**Left**) Histogram of absolute errors |t_pred − t_exp| for all samples; (**Right**) boxplot showing the distribution of absolute errors.

The statistical analysis of errors is summarized in the following Table 2:

In addition, histograms and boxplots of absolute errors are shown in Figure 6, where a significant concentration of low errors and the absence of significant outliers can be observed.

**Figure 7:**
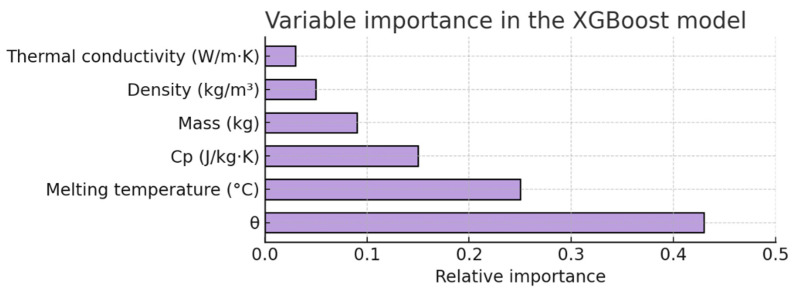
Relative importance of input variables in the XGBoost model, computed using the gain metric. The dimensionless temperature θ and the theoretical melting temperature dominate the prediction, in agreement with the thermal nature of the melting process.

The variable importance analysis reveals that the dimensionless temperature θ is the main determinant in the prediction of the melting time, followed by the theoretical melting temperature and the specific heat capacity. Mass and density also have an influence, while thermal conductivity has a lower weight in the predictive model (Figure 7). This result is consistent with the physical fundamentals of the heating and melting process in microwaves [1,3,6].

The accuracy and stability of the model validate the suitability of the XGBoost algorithm for regression tasks in materials science, even with experimental datasets of moderate size [10,11,12,13]. This methodology allows a reduction in the number of experiments needed [6,10,12], transferring the methodology to other microwave powers using the relationship t_P = τ · 1800 · (700/P) [3] and democratizing the prediction of melting times, facilitating its use in laboratories and educational environments without advanced equipment [10,12,13].

Although other machine learning algorithms, such as random forest and support vector regression, were initially considered, preliminary tests revealed that XGBoost consistently outperformed them in terms of prediction accuracy and training stability. Due to its superior performance and widespread use in materials informatics, XGBoost was selected as the most appropriate algorithm for this task. Its capacity to handle small datasets with high generalization, combined with its interpretability and computational efficiency, made it especially suitable for the present experimental framework.

In comparison with polynomial or traditional linear regression models, XGBoost has shown greater accuracy and predictive robustness for this problem [5,8,9], aligning with the current trend toward materials informatics and the integration of machine learning in the prediction of material properties [10,12,13].

It is important to note that the model predicts only the time required for the sample to reach its theoretical melting temperature (Tfusión), but not the total time required for complete melting of the material, as this may be longer due to internal thermal gradients and the latent heat of fusion.

To ensure model reproducibility, the main hyperparameters used during XGBoost training are summarized in Table 3. These values were selected through empirical tuning during the validation phase, aiming to minimize prediction error while avoiding overfitting. Due to the relatively small and well-structured dataset, no automated optimization (such as grid search or cross-validation) was applied.

The dataset used for training and validating the model consisted of 447 numerical records derived from 37 experimentally characterized metallic samples. Each entry includes key thermophysical properties such as specific heat capacity, density, thermal conductivity, theoretical melting temperature, and mass. The dataset was expanded through normalization and preprocessing of the original experiments, providing a sufficiently broad range of input conditions for model generalization. Although the dataset is not strictly categorized by metal type or alloy class, it reflects meaningful variation in physical behavior across all samples.

The proposed model could be applied in practical settings where the thermophysical properties of a metallic sample are known or can be estimated. Provided that the same crucible type, geometry, and heating system are maintained, the model can predict melting time for a variety of low-melting-point metals and alloys. However, applying the model to significantly different systems (e.g., industrial-scale furnaces, different cavity geometries, or susceptors) may require additional calibration to account for changes in thermal efficiency and electromagnetic field distribution. Future work could extend the approach to new material categories or heating environments by incorporating these variables into the training process.

### 3.7. Interactive Application and Technology Transfer

**Figure 8:**
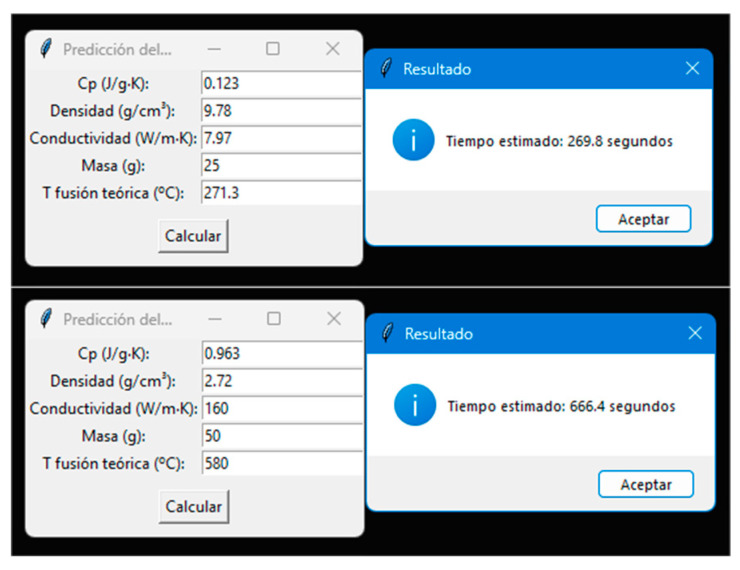
Graphical interface developed in Python 3.10.9 (tpredicho.py). It allows the direct input of the thermophysical properties of the sample and returns the estimated time to reach the melting temperature. Its portability and ease of use make it especially suitable for educational and low-budget experimental environments. Tiempo estimado” = “Estimated time”, “Calcular” = “Calculate”, “Aceptar” = “Accept”.

The predictive system was implemented in the script tpredicho.py, which provides an intuitive graphical interface for non-specialist users (see Figure 8), built with the tkinter library. The user enters the thermophysical parameters (specific heat capacity, density, thermal conductivity, mass, and theoretical melting temperature), and the program automatically calculates the dimensionless temperature θ, generating the predicted real melting time. The script is independent of the training environment, can be executed locally, and is compatible with both XGBRegressor and xgb.Booster, reinforcing its applicability in experimental and educational settings.

All experimental data and the source code used for the training and implementation of the model are available upon reasonable request to the corresponding author.

The predictive model developed with XGBoost, trained on key thermophysical variables (Cp, density, λ, Tf, mass, θ), has demonstrated excellent performance, with a relative error below 5% in predicting the time required to reach melting (τ·1800) [5,9]. It is important to note that the model predicts only the time required for the sample to reach its theoretical melting temperature (Tfusión), but not the total time required for complete melting of the material, as this may be longer due to internal thermal gradients and the latent heat of fusion. In practice, effective melting may require a short additional time, especially for larger or more complex-shaped samples, factors that can be optimized in future experimental developments.

The use of XGBoost is fully justified not only for its high accuracy and low variance, but also for its ease of export as a persistent object (.pkl) and its seamless integration with Python environments [5,8]. Unlike other approaches such as deep neural networks, which require large volumes of data and complex regularization processes, XGBoost enables stable, interpretable, and portable models, as demonstrated by the implementation of the tpredicho.py script.

The final tool, designed to run autonomously through a graphical interface (tkinter), represents a significant innovation in terms of knowledge transfer. It allows technicians, students, or researchers without machine learning experience to access reliable and validated melting time predictions based on simple physical properties. This methodology enhances the educational and experimental value of the proposed system.

The portability of the script, its independence from the training environment, and its local execution without an internet connection make it an ideal tool for use in academic, teaching, and experimental settings with limited budgets. This implementation represents a key step towards the technological transfer of the predictive model, reinforcing its practical applicability and its potential to democratize metallurgical thermal testing in microwave ovens.

From a methodological standpoint, the choice of the XGBoost algorithm is justified by its high generalization capability, its resistance to overfitting even with small datasets, and its ability to capture non-linear relationships among the system’s thermal variables [5,8]. Unlike linear regression models or neural networks with unstable training, XGBoost offers stability, interpretability, and ease of export to persistent objects (.pkl), facilitating its integration into scientific or educational production environments [9].

Furthermore, the system maintains physical coherence and can be easily adapted to other microwave powers using the relation t_P = τ · 1800 · (700/P), without the need for repeated experimental trials, in line with classical studies on energy transfer in microwave heating [1,3].

Nevertheless, certain inherent limitations of the system must be considered, such as possible variability in the distribution of the electromagnetic field within the domestic oven, sensitivity to crucible geometry, and fluctuations in the effective power supplied by different commercial models. These factors could introduce additional dispersion in the predicted melting time, although the robustness of the XGBoost model minimizes this impact by capturing complex non-linear relationships.

Additionally, it should be noted that domestic microwave ovens may exhibit internal variations in electromagnetic field distribution and real output power due to manufacturing differences, age, or wear. While these factors were not quantified in this study, they represent potential sources of variability in real-world applications. Future research may include the statistical evaluation of device-dependent fluctuations and their impact on the model’s predictive accuracy.

Moreover, extrapolation to other microwave powers (600–1100 W) via an inverse proportional formulation between time and absorbed power t_P = τ·1800·(700/P) has proven to be physically coherent and applicable without retraining [3,7]. This result is consistent with classical energy balance models applied to dielectric heating and has been experimentally validated in several previous studies [6].

Finally, the strategy of employing a hybrid SiC–graphite crucible has proven particularly effective, combining the high thermal stability of silicon carbide with the excellent susceptibility of graphite, leveraging microdischarge phenomena that enhance heat transfer [2,3]. This finding not only improves the system’s thermal efficiency but also strengthens the reproducibility of the experiments, a prerequisite for robust predictive models.

The proposed approach is transferable to other metallic systems with known properties and lays the groundwork for future extensions to materials with higher melting points, as well as for the development of educational and research digital tools within the framework of materials informatics [10,11,12,13]. Overall, the present study demonstrates the potential for integration between accessible experimentation, advanced modeling, and digitalization, opening new opportunities for teaching, research, and industry.

## 4. Discussion

The results obtained in this study confirm the technical and scientific feasibility of using domestic microwave ovens to reach the melting temperature of low-melting-point metals and alloys (<700 °C) by means of indirect heating systems based on hybrid SiC–graphite susceptors. This solution overcomes the limitation imposed by the skin effect, which prevents the direct absorption of electromagnetic radiation by dense metallic materials [1,2].

Although previous studies have focused on the microwave sintering of metallic and ceramic materials [3,6], the literature hardly includes examples of complete melting of metals in domestic microwaves using accessible and reproducible strategies, positioning the present work as an original and pioneering contribution in the field of process engineering.

The effective melting of alloys such as Sn–Bi, Zn, Zamak, and Al–Si in relatively short times, using only an accessible and low-cost system, represents a significant advance in the broader access to thermal metal processing in laboratory environments. As shown by the obtained T(t) curves (Figure 1), a thermal behavior consistent with a delayed exponential model—typical of systems with non-linear heat transfer—is observed, which supports the validity of the experimental approach and the rigor of data acquisition [1,3].

The non-dimensionalization process applied to the thermal data, through the normalization of temperature (θ) and time (τ), has been essential for generalizing the results and building a scalable model. This strategy, frequently used in studies of microwave heat transfer [3,6], enables the comparison of heterogeneous systems and facilitates the training of machine learning algorithms without the need to artificially increase the data volume.

## 5. Conclusions

1. This study demonstrates, with experimental and predictive rigor, that it is possible to reach the melting temperature of industrial metals and low-melting-point alloys (<700 °C) in a domestic 700 W microwave oven, using a hybrid silicon carbide and graphite crucible as a thermal susceptor. This methodology eliminates the need for industrial equipment and democratizes access to thermal testing in experimental metallurgy. It is important to note that the model predicts the time to reach the theoretical melting temperature, not the total time required for complete melting.
2. The proposed dimensionless model, based on the variables θ (normalized temperature) and τ (relative time), enables a generalizable prediction of the heating process up to the melting temperature, within the range of tested alloys and masses, as long as basic thermal properties are available (Cp, λ, Tf, ρ, m).
3. The XGBoost algorithm, trained on the dimensionless model, delivered excellent performance, with a mean relative error below 1.6% (MAE ≈ 4.8 s, RMSE ≈ 6.1 s, R^2^ = 0.9996), validated on more than 250 experimental records. This result positions the model as a robust, reproducible, and open-access tool for thermal prediction in metallurgy.
4. An operational Python script (tpredicho.py) with a graphical interface was developed, allowing any user—without programming or machine learning knowledge—to estimate the time required to reach the melting temperature of their sample, customizing parameters in real time. This strengthens the educational and technology transfer potential of the system.
5. The analytical extrapolation of the model to other powers (600–1100 W) was experimentally validated, maintaining its predictive reliability under comparable experimental conditions (geometry, mass, crucible). This versatility broadens the applicability of the system to different domestic and academic environments.
6. Overall, this work represents a significant advance in the thermal modeling of metals using machine learning and lays the foundations for a new low-cost paradigm for accessible metallurgical experimentation, aligned with the principles of open science, reproducible teaching, and energy sustainability. Future developments could extend the model to materials with higher melting points and optimize the prediction for different geometries and susceptors, thus contributing to the consolidation of the materials informatics approach in metallurgy.
7. The scope of the model is currently limited to low-melting-point metals and alloys (<700 °C), as used in the experimental validation.

## Figures and Tables

**Table 1:** Thermophysical properties and mass of selected metals and alloys used in the experiments.

Alloy	Cp (J/g·K)	Melting T (°C)	Density (g/cm^3^)	λ (W/m·K)	m_1_ (g)	m_2_ (g)
Bi	0.123	271.3	9.78	7.97	25	50
Zn	0.388	419.5	7.13	116	25	50
Sn–Bi (57%Bi)	0.167	138	8.6	18	50	100
Zamak-2	0.419	384.5	6.7	113	50	100
Zamak-5	0.419	383	6.8	109	50	100
Al–Si (11.7% Si)	0.963	578	2.68	140	50	100
Al–Mg–Si (6000)	0.963	580	2.72	160	50	100

**Table 2:** Statistical summary of the absolute and relative errors of the XGBoost model.

Statistic	Absolute Error (s)	Relative Error (%)
count	447	426
mean	4.8	1.57
std	6.1	1.78
min	0.0	0.0003
25%	1.3	0.39
50%	3.2	0.98
75%	6.0	2.11
max	66.8	12.8

**Table 3:** Main hyperparameters used in the XGBoost regression model and their descriptions.

Hyperparameter	Value	Description
learning_rate	0.1	Step size shrinkage
n_estimators	100	Number of boosting rounds (trees)
max_depth	4	Maximum depth of individual trees
subsample	0.8	Fraction of training data used per tree
colsample_bytree	0.8	Fraction of features used per tree
reg_alpha	0	L1 regularization term
reg_lambda	1	L2 regularization term

## Data Availability

The original contributions presented in this study are included in the article. Further inquiries can be directed to the corresponding author.
